# Supplementary material for: Pyruvate Dehydrogenase Kinase 1 inhibition mediated oxidative phosphorylation enhancement in cartilage promotes osteoarthritis progression
Source: BMC Musculoskelet Disord. 2023 Jul 20;24:597. doi: 10.1186/s12891-023-06585-6 (PMC10357736; doi:10.1186/s12891-023-06585-6)
Supplement: Supplementary file 1 — Additional file 1. [file 12891_2023_6585_MOESM1_ESM.zip › BP.Gsea.1653623667859/GOBP_MITOCHONDRIAL_RESPIRATORY_CHAIN_COMPLEX_ASSEMBLY.html]

Details for gene set GOBP\_MITOCHONDRIAL\_RESPIRATORY\_CHAIN\_COMPLEX\_ASSEMBLY[GSEA]

|  || Dataset | 55457\_collapsed\_to\_symbols.cls.cls #OA\_versus\_normal.cls.cls #OA\_versus\_normal\_repos |
| Phenotype | cls.cls#OA\_versus\_normal\_repos |
| Upregulated in class | OA |
| GeneSet | GOBP\_MITOCHONDRIAL\_RESPIRATORY\_CHAIN\_COMPLEX\_ASSEMBLY |
| Enrichment Score (ES) | 0.64555174 |
| Normalized Enrichment Score (NES) | 2.750717 |
| Nominal p-value | 0.0 |
| FDR q-value | 0.0 |
| FWER p-Value | 0.0 |
Table: GSEA Results Summary

  

Fig 1: Enrichment plot: GOBP\_MITOCHONDRIAL\_RESPIRATORY\_CHAIN\_COMPLEX\_ASSEMBLY      
 Profile of the Running ES Score & Positions of GeneSet Members on the Rank Ordered List

  

| SYMBOL | TITLE | RANK IN GENE LIST | RANK METRIC SCORE | RUNNING ES | CORE ENRICHMENT || 1 | COX17 | cytochrome c oxidase copper chaperone COX17 [Source:HGNC Symbol;Acc:HGNC:2264] | 64 | 0.966 | 0.0311 | Yes |
| 2 | NDUFA8 | NADH:ubiquinone oxidoreductase subunit A8 [Source:HGNC Symbol;Acc:HGNC:7692] | 67 | 0.961 | 0.0668 | Yes |
| 3 | SAMM50 | SAMM50 sorting and assembly machinery component [Source:HGNC Symbol;Acc:HGNC:24276] | 99 | 0.915 | 0.0985 | Yes |
| 4 | TMEM186 | transmembrane protein 186 [Source:HGNC Symbol;Acc:HGNC:24530] | 128 | 0.876 | 0.1290 | Yes |
| 5 | NDUFS3 | NADH:ubiquinone oxidoreductase core subunit S3 [Source:HGNC Symbol;Acc:HGNC:7710] | 177 | 0.836 | 0.1565 | Yes |
| 6 | NDUFA1 | NADH:ubiquinone oxidoreductase subunit A1 [Source:HGNC Symbol;Acc:HGNC:7683] | 194 | 0.826 | 0.1860 | Yes |
| 7 | NDUFA13 | NADH:ubiquinone oxidoreductase subunit A13 [Source:HGNC Symbol;Acc:HGNC:17194] | 236 | 0.795 | 0.2125 | Yes |
| 8 | NDUFA10 | NADH:ubiquinone oxidoreductase subunit A10 [Source:HGNC Symbol;Acc:HGNC:7684] | 243 | 0.788 | 0.2415 | Yes |
| 9 | NDUFB6 | NADH:ubiquinone oxidoreductase subunit B6 [Source:HGNC Symbol;Acc:HGNC:7701] | 262 | 0.779 | 0.2691 | Yes |
| 10 | NDUFAF3 | NADH:ubiquinone oxidoreductase complex assembly factor 3 [Source:HGNC Symbol;Acc:HGNC:29918] | 285 | 0.761 | 0.2958 | Yes |
| 11 | TMEM126B | transmembrane protein 126B [Source:HGNC Symbol;Acc:HGNC:30883] | 295 | 0.753 | 0.3232 | Yes |
| 12 | COA1 | cytochrome c oxidase assembly factor 1 [Source:HGNC Symbol;Acc:HGNC:21868] | 426 | 0.695 | 0.3390 | Yes |
| 13 | NDUFB3 | NADH:ubiquinone oxidoreductase subunit B3 [Source:HGNC Symbol;Acc:HGNC:7698] | 483 | 0.667 | 0.3596 | Yes |
| 14 | COX16 | cytochrome c oxidase assembly factor COX16 [Source:HGNC Symbol;Acc:HGNC:20213] | 498 | 0.660 | 0.3831 | Yes |
| 15 | NDUFS4 | NADH:ubiquinone oxidoreductase subunit S4 [Source:HGNC Symbol;Acc:HGNC:7711] | 531 | 0.647 | 0.4047 | Yes |
| 16 | NDUFA3 | NADH:ubiquinone oxidoreductase subunit A3 [Source:HGNC Symbol;Acc:HGNC:7686] | 550 | 0.641 | 0.4273 | Yes |
| 17 | COA3 | cytochrome c oxidase assembly factor 3 [Source:HGNC Symbol;Acc:HGNC:24990] | 597 | 0.625 | 0.4470 | Yes |
| 18 | NDUFA2 | NADH:ubiquinone oxidoreductase subunit A2 [Source:HGNC Symbol;Acc:HGNC:7685] | 878 | 0.549 | 0.4457 | Yes |
| 19 | NDUFB5 | NADH:ubiquinone oxidoreductase subunit B5 [Source:HGNC Symbol;Acc:HGNC:7700] | 902 | 0.542 | 0.4641 | Yes |
| 20 | TACO1 | translational activator of cytochrome c oxidase I [Source:HGNC Symbol;Acc:HGNC:24316] | 923 | 0.536 | 0.4826 | Yes |
| 21 | NDUFC2 | NADH:ubiquinone oxidoreductase subunit C2 [Source:HGNC Symbol;Acc:HGNC:7706] | 929 | 0.532 | 0.5020 | Yes |
| 22 | NDUFA9 | NADH:ubiquinone oxidoreductase subunit A9 [Source:HGNC Symbol;Acc:HGNC:7693] | 943 | 0.529 | 0.5208 | Yes |
| 23 | NDUFB4 | NADH:ubiquinone oxidoreductase subunit B4 [Source:HGNC Symbol;Acc:HGNC:7699] | 968 | 0.525 | 0.5385 | Yes |
| 24 | NDUFB7 | NADH:ubiquinone oxidoreductase subunit B7 [Source:HGNC Symbol;Acc:HGNC:7702] | 1033 | 0.510 | 0.5526 | Yes |
| 25 | NDUFB1 | NADH:ubiquinone oxidoreductase subunit B1 [Source:HGNC Symbol;Acc:HGNC:7695] | 1055 | 0.506 | 0.5698 | Yes |
| 26 | NDUFS7 | NADH:ubiquinone oxidoreductase core subunit S7 [Source:HGNC Symbol;Acc:HGNC:7714] | 1071 | 0.501 | 0.5873 | Yes |
| 27 | NDUFC1 | NADH:ubiquinone oxidoreductase subunit C1 [Source:HGNC Symbol;Acc:HGNC:7705] | 1091 | 0.499 | 0.6045 | Yes |
| 28 | NDUFS8 | NADH:ubiquinone oxidoreductase core subunit S8 [Source:HGNC Symbol;Acc:HGNC:7715] | 1208 | 0.474 | 0.6131 | Yes |
| 29 | NDUFS1 | NADH:ubiquinone oxidoreductase core subunit S1 [Source:HGNC Symbol;Acc:HGNC:7707] | 1229 | 0.471 | 0.6291 | Yes |
| 30 | NDUFB11 | NADH:ubiquinone oxidoreductase subunit B11 [Source:HGNC Symbol;Acc:HGNC:20372] | 1603 | 0.413 | 0.6155 | Yes |
| 31 | SURF1 | SURF1 cytochrome c oxidase assembly factor [Source:HGNC Symbol;Acc:HGNC:11474] | 1829 | 0.381 | 0.6122 | Yes |
| 32 | AIFM1 | apoptosis inducing factor mitochondria associated 1 [Source:HGNC Symbol;Acc:HGNC:8768] | 1838 | 0.380 | 0.6258 | Yes |
| 33 | NDUFS5 | NADH:ubiquinone oxidoreductase subunit S5 [Source:HGNC Symbol;Acc:HGNC:7712] | 2267 | 0.330 | 0.6048 | Yes |
| 34 | TTC19 | tetratricopeptide repeat domain 19 [Source:HGNC Symbol;Acc:HGNC:26006] | 2394 | 0.315 | 0.6067 | Yes |
| 35 | SDHAF3 | succinate dehydrogenase complex assembly factor 3 [Source:HGNC Symbol;Acc:HGNC:21752] | 2409 | 0.313 | 0.6173 | Yes |
| 36 | NDUFB2 | NADH:ubiquinone oxidoreductase subunit B2 [Source:HGNC Symbol;Acc:HGNC:7697] | 2460 | 0.307 | 0.6249 | Yes |
| 37 | FASTKD3 | FAST kinase domains 3 [Source:HGNC Symbol;Acc:HGNC:28758] | 2473 | 0.306 | 0.6353 | Yes |
| 38 | NUBPL | nucleotide binding protein like [Source:HGNC Symbol;Acc:HGNC:20278] | 2569 | 0.295 | 0.6389 | Yes |
| 39 | NDUFB8 | NADH:ubiquinone oxidoreductase subunit B8 [Source:HGNC Symbol;Acc:HGNC:7703] | 2623 | 0.288 | 0.6456 | Yes |
| 40 | NDUFS2 | NADH:ubiquinone oxidoreductase core subunit S2 [Source:HGNC Symbol;Acc:HGNC:7708] | 3072 | 0.243 | 0.6197 | No |
| 41 | NDUFA6 | NADH:ubiquinone oxidoreductase subunit A6 [Source:HGNC Symbol;Acc:HGNC:7690] | 3834 | 0.173 | 0.5669 | No |
| 42 | SDHAF1 | succinate dehydrogenase complex assembly factor 1 [Source:HGNC Symbol;Acc:HGNC:33867] | 4303 | 0.128 | 0.5353 | No |
| 43 | NDUFAF1 | NADH:ubiquinone oxidoreductase complex assembly factor 1 [Source:HGNC Symbol;Acc:HGNC:18828] | 4388 | 0.122 | 0.5333 | No |
| 44 | NDUFAB1 | NADH:ubiquinone oxidoreductase subunit AB1 [Source:HGNC Symbol;Acc:HGNC:7694] | 4445 | 0.118 | 0.5333 | No |
| 45 | UQCC1 | ubiquinol-cytochrome c reductase complex assembly factor 1 [Source:HGNC Symbol;Acc:HGNC:15891] | 5281 | 0.049 | 0.4701 | No |
| 46 | UQCRFS1 | "ubiquinol-cytochrome c reductase, Rieske iron-sulfur polypeptide 1 [Source:HGNC Symbol;Acc:HGNC:12587]" | 5509 | 0.032 | 0.4536 | No |
| 47 | OXA1L | OXA1L mitochondrial inner membrane protein [Source:HGNC Symbol;Acc:HGNC:8526] | 5974 | -0.003 | 0.4176 | No |
| 48 | NDUFAF4 | NADH:ubiquinone oxidoreductase complex assembly factor 4 [Source:HGNC Symbol;Acc:HGNC:21034] | 6087 | -0.011 | 0.4093 | No |
| 49 | NDUFAF5 | NADH:ubiquinone oxidoreductase complex assembly factor 5 [Source:HGNC Symbol;Acc:HGNC:15899] | 6167 | -0.018 | 0.4038 | No |
| 50 | NDUFA5 | NADH:ubiquinone oxidoreductase subunit A5 [Source:HGNC Symbol;Acc:HGNC:7688] | 6789 | -0.062 | 0.3578 | No |
| 51 | COA4 | cytochrome c oxidase assembly factor 4 homolog [Source:HGNC Symbol;Acc:HGNC:24604] | 7460 | -0.115 | 0.3099 | No |
| 52 | BCS1L | "BCS1 homolog, ubiquinol-cytochrome c reductase complex chaperone [Source:HGNC Symbol;Acc:HGNC:1020]" | 7578 | -0.125 | 0.3055 | No |
| 53 | DMAC2 | distal membrane arm assembly component 2 [Source:HGNC Symbol;Acc:HGNC:25496] | 8347 | -0.182 | 0.2525 | No |
| 54 | NDUFAF7 | NADH:ubiquinone oxidoreductase complex assembly factor 7 [Source:HGNC Symbol;Acc:HGNC:28816] | 10077 | -0.328 | 0.1301 | No |
| 55 | TAFAZZIN | "tafazzin, phospholipid-lysophospholipid transacylase [Source:HGNC Symbol;Acc:HGNC:11577]" | 10429 | -0.364 | 0.1163 | No |
| 56 | SCO2 | synthesis of cytochrome C oxidase 2 [Source:HGNC Symbol;Acc:HGNC:10604] | 11410 | -0.488 | 0.0583 | No |
| 57 | TFAM | "transcription factor A, mitochondrial [Source:HGNC Symbol;Acc:HGNC:11741]" | 12175 | -0.637 | 0.0226 | No |
| 58 | MT-ND5 | mitochondrially encoded NADH:ubiquinone oxidoreductase core subunit 5 [Source:HGNC Symbol;Acc:HGNC:7461] | 12743 | -0.906 | 0.0122 | No |
Table: GSEA details [plain text format]

  

Fig 2: GOBP\_MITOCHONDRIAL\_RESPIRATORY\_CHAIN\_COMPLEX\_ASSEMBLY      
 Blue-Pink O' Gram in the Space of the Analyzed GeneSet

  

Fig 3: GOBP\_MITOCHONDRIAL\_RESPIRATORY\_CHAIN\_COMPLEX\_ASSEMBLY: Random ES distribution      
 Gene set null distribution of ES for **GOBP\_MITOCHONDRIAL\_RESPIRATORY\_CHAIN\_COMPLEX\_ASSEMBLY**
